# Drug-releasing mesenchymal cells strongly suppress B16 lung metastasis in a syngeneic murine model

**DOI:** 10.1186/s13046-015-0200-3

**Published:** 2015-08-13

**Authors:** Augusto Pessina, Carlo Leonetti, Simona Artuso, Anna Benetti, Enrico Dessy, Luisa Pascucci, Daniela Passeri, Augusto Orlandi, Angiola Berenzi, Arianna Bonomi, Valentina Coccè, Valentina Ceserani, Anna Ferri, Marta Dossena, Pietro Mazzuca, Emilio Ciusani, Piero Ceccarelli, Arnaldo Caruso, Nazario Portolani, Francesca Sisto, Eugenio Parati, Giulio Alessandri

**Affiliations:** Department of Biomedical, Surgical and Dental Sciences, University of Milan, Via Pascal 36, Milan, 20133 Italy; Experimental Chemotherapy Laboratory, Regina Elena National Cancer Institute, Rome, Italy; Department of Clinical and Experimental Sciences, Institute of Pathological Anatomy, University of Brescia, Brescia, Italy; Department of Molecular and Translational Medicine, University of Brescia, Brescia, Italy; Department of Veterinary Medicine, University of Perugia, Perugia, Italy; Department of Biopathology and Image Diagnostics, Anatomic Pathology Institute, University of Rome ‘Tor Vergata’, Rome, Italy; Cellular Neurobiology Laboratory, Department of Cerebrovascular Diseases, IRCCS Neurological Institute C. Besta, Milan, Italy; Department of Microbiology, Brescia University, Brescia, Italy; Laboratory of Clinical Pathology and Neurogenetic Medicine, Fondazione IRCCS Neurological Institute Carlo Besta, Milan, Italy; Department of Medical and Surgical Sciences, University of Brescia, Brescia, Italy

**Keywords:** Mesenchymal stromal cells, Lung metastasis, Paclitaxel, SDF-1, B16 melanoma, Drug-delivery

## Abstract

**Background:**

Mesenchymal stromal cells (MSCs) are considered an important therapeutic tool in cancer therapy. They possess intrinsic therapeutic potential and can also be *in vitro* manipulated and engineered to produce therapeutic molecules that can be delivered to the site of diseases, through their capacity to home pathological tissues. We have recently demonstrated that MSCs, upon *in vitro* priming with anti-cancer drug, become drug-releasing mesenchymal cells (Dr-MCs) able to strongly inhibit cancer cells growth.

**Methods:**

Murine mesenchymal stromal cells were loaded with Paclitaxel (Dr-MCsPTX) according to a standardized procedure and their ability to inhibit the growth of a murine B16 melanoma was verified by *in vitro* assays. The anti-metastatic activity of Dr-MCsPTX was then studied in mice injected i.v. with B16 melanoma cells that produced lung metastatic nodules. Lung nodules were counted under a dissecting stereomicroscope and metastasis investigated by histological analysis.

**Results:**

We found that three i.v. injections of Dr-MCsPTX on day 5, 10 and 15 after tumor injection almost completely abolished B16 lung metastasis. Dr-MCsPTX arrested into lung by interacting with endothelium and migrate toward cancer nodule through a complex mechanism involving primarily mouse lung stromal cells (mL-StCs) and SDF-1/CXCR4/CXCR7 axis.

**Conclusions:**

Our results show for the first time that Dr-MCsPTX are very effective to inhibit lung metastasis formation. Actually, a cure for lung metastasis in humans is mostly unlikely and we do not know whether a therapy combining engineered MSCs and Dr-MCs may work synergistically. However, we think that our approach using Dr-MCs loaded with PTX may represent a new valid and additive therapeutic tool to fight lung metastases and, perhaps, primary lung cancers in human.

**Electronic supplementary material:**

The online version of this article (doi:10.1186/s13046-015-0200-3) contains supplementary material, which is available to authorized users.

## Background

Adult mesenchymal stromal cells (MSCs) are a suitable cell source for cell-based therapies [1]. MSCs possess intrinsic therapeutic potential [2], however, they can be *in vitro* manipulated and engineered to produce therapeutic molecules [3–6] that can be delivered to the site of disease, through their capacity to home pathological tissues [7–9].

Despite the molecular mechanisms involved in MSCs homing, the number of cells that reach a disease area may depend on the route of their administration [10, 11]. After intravenous (i.v) injection most of the cells remain trapped in draining organs such as lung, kidney and liver [12] and this may drastically limit the number of MSCs that can reach pathological area in other organs. However, lung, besides its draining capacity, has been shown a promise tissue target for cell-based therapies [13, 14].

Previous data from our group have shown that MSCs can acquire strong anti-cancer activity upon *in vitro* exposure to very high dose of chemotherapeutic agent such as Paclitaxel (PTX). In particular, we have shown that *in vivo* the co-injection as well as the intra-tumor injection of MSCs loaded with PTX strong inhibit tumor growth [15, 16]. MSCs become drug-releasing mesenchymal cells (Dr-MCs) because they are able to uptake and release the drug and thus kill tumor cells when located nearby. This property seems present not only in MSCs derived from bone marrow (BM) but also in those derived from adipose tissue [17] or in fibroblasts [18] and even in monocytes [19].

In order to expand our previous work, we here investigated the capacity of Dr-MCs loaded with PTX to inhibit lung metastasis induced by i.v. injection of B16 melanoma cells in syngeneic C57Bl6 mice. The Dr-MCs here used were the syngeneic SR4987 a MSC line developed from BM of BDF/1 mice [20]. We found that SR4987 loaded with PTX (SR4987PTX) i.v. injected after tumor cells administration, abolished almost all lung metastasis. We demonstrated that SR4987PTX once in lung, accumulate nearby metastatic foci through a complex mechanism involving resident mouse lung stromal cells (mL-StCs), Stromal-Derived Factor 1 (SDF-1) and its receptors CXCR4/CXCR7. We propose to use Dr-MCs loaded with PTX as new therapeutic approach for lung metastasis.

## Materials and methods

### Cells

The murine melanoma cell line B16 [21] and MOLT-4 (human acute T-lymphoblastic leukemia) [22] were provided by Centro Substrati Cellulari, ISZLER (Brescia, Italy). B16 cells were maintained in RPMI 1640 medium (Euroclone, UK) supplemented with 10 % fetal bovine serum (FBS) (LONZA Walkersville MD USA). MOLT-4 cells were cultured in complete IMDM +10 % FBS. The Mesenchymal stromal cell line SR4987 was established from a long-term BM-derived cell culture of BDF/1 mice (ATCC CRL-2028) [23]. SR4987 cells were cultured in IMDM supplemented with 5 % FBS. SR4987 are positive for stem cell marker Vimentin, CD44+, CD73+, CD105+, CD106+, Sca-1+, CD34+, and CD45+ and have the capacity to differentiate into osteoblasts and chondrocytes [23]. SR4987 cells were also transduced with green fluorescent protein (GFP) using lentiviral vector ( pCCLsin.PPThPGK.GFPpre) as previously described [15].

Human lung-derived microvascular endothelial cells (L-MECs) were obtained from Dr. Arnaldo Caruso (Laboratory of Microbiology, University of Brescia, Brescia, Italy). L-MECs were routinely maintained in endothelial basal growth medium (EBM-GM) (EGM bullet kit, LONZA Walkersville MD USA) on Collagen + Fibronectin coated T25 flasks.

Mouse lung stromal cells (mL-StCs) were freshly isolated from C57Bl6 mice by using 0.25 % collagenase D (Boehringer Mannheim Germany). The adherent mL-StCs were cultured in IMDM +5 % FBS and analyzed for mesenchymal markers CD44, CD29 and CD90 (all purchased from Abcam Cambridge UK, mouse MSCs panel) to confirm their mesenchymal origin.

### PTX priming of SR4987 cells

PTX was purchased from Adipogen (Vinci-Biochem, Italy). The activity of PTX on SR4987 cells was determined in a 24 h (cytotoxicity test) and in a 7 day MTT assay (anti-proliferative test) as previously described [24]. Priming of SR4987 with PTX (SR4987PTX) was carried out using PTX at 2,000 ng/ml as previously described [15].

### *In vitro* antitumor assay

The effect of conditioned medium (CM) from SR4987 and SR4987PTX cells on B16 proliferation was evaluated by MTT assay [24]. The inhibitory concentrations (IC_50_ and IC_90_) were determined according to the Reed and Muench formula [25]. The antitumor activity of CM from SR4987PTX cells was compared to that of pure PTX and expressed as PTX equivalent concentration (PEC) [15]. The PTX released by a single cells was calculated dividing PEC for the number of cells seeded and expressed as pg/cell. To establish the optimal dose of SR4987PTX to inject, co-cultures with B16 cells were made by using transwell inserts (0.4 μm pore size) (Becton Dickinson, USA) [15, 16].

### Rosette adherence assay and Transmission electron microscopy (TEM) analysis

To study the interaction between B16 cells and SR4987, a rosette test was performed [16]. B16 were mixed in a conical tube with SR4987 or SR4987PTX cells, in 500 μl of IMDM + 5 % FBS (ratio B16/SR4987 5:1). After 24 h of incubation at 37 °C in air + 5 % CO_2_, 20 μl of cells were collected by a micropipette and then transferred on a slide to evaluate rosette formation under an inverted microscope. Rosette were then analyzed by TEM as previously described [16].

### Adhesion assay

Adhesion of SR4987 and SR4987PTX to L-MECs and to mL-StCs were performed following a previously described procedure [16]. L-MECs and mL-StCs monolayers were stimulated by adding B16 derived CM (B16-CM), tumor necrosis factor-*alpha* (TNFα; 10 ng/ml; Sigma Chemical) and their combination for 24 h. L-MECs and mL-StCs were allowed to interact for 30’ with SR4987 and SR4987PTX (10^4^ cells/well) in IMDM +0.2 % BSA. Each test was run in quadruplicate (see Additional file 1).

### Migration assay

Transwell supports were used to test spontaneous migration and chemotaxis of SR4987 and SR4987PTX. Migration assay was performed as previously described [26]. Test samples consisting in: mouse SDF-1 (1-100 ng/ml) (R&D system), TNFα (1-50 ng/ml), B16-CM (1:1-1:100 dilutions) mL-StCs-CM (1:1-1:100 dilutions), CM derived from mL-StCs primed for 24 h either with B16-CM (1:1) (mL-StCs-B16-CM), TNFα 10 ng/ml (mL-StCs-TNFα-CM) or B16-CM + TNFα (mL-StCs-B16-TNFα-CM), were placed in the lower compartment. The blocking effect of monoclonal antibodies (mAbs) anti mouse SDF-1 (R & D System), as well control mouse IgG, (0.01-1ug/ml) was tested by adding mAbs to medium containg SDF-1 or to CMs. To block SDF-1 receptors CXCR4/CXCR7, SR4987 and SR4987PTX (5×10^5^) were pre-incubated for 1 h at 37 °C in IMDM + 0.2 % BSA in presence of different concentration of AMD3100 (0.1-10 μM) (purchased from Sigma). Each determination was done in duplicate (see Additional file 1).

### Immunoassay for mouse SDF-1

CM samples recovered from 48 h cultured SR4987, SR4987PTX, B16 cells, or from mL-StCs stimulated or not with B16-CM, TNFα and B16-CM + TNFα were analyzed for the presence of SDF-1 using an Elisa kit (R&D System). The value of SDF-1 detected in the CM were normalized for the same number of cells counted at the end of incubation.

### Flow Cytometry and PCR

The expression of Sca-1, CXCR4 and CXCR7 on SR4987 was determind by flow cytometry (FC) using the following antibodies: anti-Sca-1 FITC, anti-CXCR4 (ab1670) and anti-CXCR7 (all purchased from Abcam Cambridge UK). 20,000 events were acquired for each analysis using Epics “XL-MLC” (Beckman Coulter, USA) and histogram elaboration was performed with EXPO 32 software.

For the analysis of mRNA levels 1000 ng of total RNA isolated using the RNeasy kit (Qiagen) was reverse-transcribed using iScript cDNA Synthesis Kit (Bio-Rad Laboratories). Triplicate polymerase chain reactions were carried out on an CFX 96 Touch Real Time PCR Detection System (Bio-Rad Laboratories). Relative gene expression was calculated by a comparative method (2^-ΔΔCt^) using GAPDH as an housekeeping gene. Primers sequences were designed using Primer3 software.

### *In vivo* experiments and Immunohistochemistry

Six-to eight-week old male C57BL/6 mice were purchased from Charles River (Calco, Milan, Italy). All the animal experiments were performed at the Animal Facility of Regina Elena National Cancer Institute in Rome Italy following directive 2010/63/EU. Healthy mice (2 each group) receiving i.v. injection of saline, 10^6^ SR4987GFP and 10^6^ SR4987GFP-PTX. Fluorescent cells were used to confirm their capacity to arrest into the lung. Mice were sacrificed at 6 h, 24 h and 48 h after treatments and lungs were minced and digested by collagenase (for 2 h at 37 °C) to obtain a cell suspension that was rapidly analyzed under Fluorescent microscopy. To study the anti-metastatic activity of SR4987PTX, mice were injected i.v. with 2.5 × 10^5^ B16 melanoma cells /0.2 ml. On day 5, after B16 injection, mice (12 each group) were treated i.v. with 0.2 ml saline (controls), PTX 10 mg/Kg, SR4987 (10^5^/0.2 ml) and SR4987PTX at 10^5^ and 5×10^4^/0.2 ml. Treatments were repeated on day 10 and 15. On day 21 after B16 injection, mice were euthanized, lungs removed and fixed in Bouin’s solution. Lung nodules were counted under a dissecting stereomicroscope. Metastasis were also investigated by histology using Hematoxylin and Eosin (HE) and by Fontana-Masson staining to detect micrometastasis [26]. To investigate the capacity of SR4987 and SR4987PTX to home lung B16 nodules, mice (3 each group) were injected i.v. with 10^6^ B16 cells. Upon 14 days mice were i.v. treated with saline (controls), PTX (10 mg/kg), SR4987 (10^5^) and SR4987PTX (10^5^) and sacrificed 48 h after treatments. The lungs were processed by immunohistochemistry for Sca-1 + cells detection by using rabbit monoclonal anti-Sca1/Ly6A/E antibody (1:150, Abcam) and polyclonal anti-SDF-1 (Santa Cruz Biotechnology Inc USA) for SDF-1 expression according to standard protocols [26, 27]. The number of Sca-1^+^cells was evaluated at high power field (HPF), 400X magnification. At least 10 fields for each case were randomly evaluated with variability less than 5 %.

### Statistical analysis

Data are expressed as the mean ± standard deviation (SD). Differences between mean values were evaluated according to Student’s t-test performed by GRAPHPADINSTAT program (GraphPad Software Inc., San Diego, CA, USA). p values < 0.05 were considered statistically significant.

## Results

### SR4987 localized in the lung after i.v. injection

In preliminary experiments we verified if mouse SR4987 and SR4987PTX cells localized in the lung after i.v injection. At this purpose, GFP transfected SR4987 cells (SR4987GFP) were prepared [15]. At 6 h, 24 h, and 48 h following cells injection, mice were sacrificed and lungs analyzed for the presence of SR4987GFP. Both groups of mice treated with cells retained in the lung significant amount of GFP+ cells until 48 h upon injection. Maximal presence of cells was observed between 6 and 24 h. The viability of the GFP+ cells arrested in lungs was confirmed by culturing the cells obtained after lungs digestion. The presence of injected cells in the lung were also ascertained by evaluating Sca-1, a marker that is considered specific for murine MSCs [28] and highly expressed on SR4987 (Additional file 2: Figure S2A). A significant presence of Sca-1+ cells in both SR4987 and SR4987PTX treated mice were found, while rare Sca-1+ cells were detected in untreated mice (Additional file 2: Figure S2B) by indicating that physiological Sca-1 positive cells with bone marrow origin is rarely seen.

### SR4987PTX bind B16 melanoma cells and inhibit their in vitro proliferation

In previous studies we have shown that SR4987 cells behaved as Dr-MCs since are very resistant to PTX cytotoxicity and able to uptake/release the drug [15, 16]. The quantification of PTX released by SR4987PTX was performed by testing the anti-proliferative activity of CM (SR4987PTX-CM) on MOLT-4, a leukemia cell line very sensitive to PTX [29]. SR4987PTX release around 0.34 ± 0.01 pg and incorporate around 2.5 ± 0.05 pg of PTX/cell in 24 h [15]. We found that SR4987PTX-CM inhibited B16 proliferation in a dose-dependent manner, with an IC50 at dilution 1/9.98 ± 2.4 corresponding at 7.18 ± 4.99 ng/ml PTX (all results are summarized in Additional file 2: Figure S3). To establish the dose of SR4987PTX cells to inject in vivo, we co-cultured SR4987PTX and B16 cells at different ratio using trans-well inserts. We found that the optimal dose/ratio of SR4987PTX/B16 was around 1/5 that inhibited B16 melanoma proliferation of 70 % (Additional file 2: Figure S4).

SR4987 and SR4987PTX were also able to bind B16 cells by a rosette formation assay. The ultrastructure analysis of the rosette showed the presence of junctional-like structure and even a gap-like junction between SR4987 and B16 cells. At TEM, the potent cytotoxic activity of SR4987PTX on B16 cells was very evident; most of the B16 cells bound to SR4987PTX were apoptotic and necrotic (Additional file 2: Figure S5).

### *In vivo* systemic administration of SR4987 PTX suppresses B16 lung metastasis in syngeneic mice

**Fig. 1:**
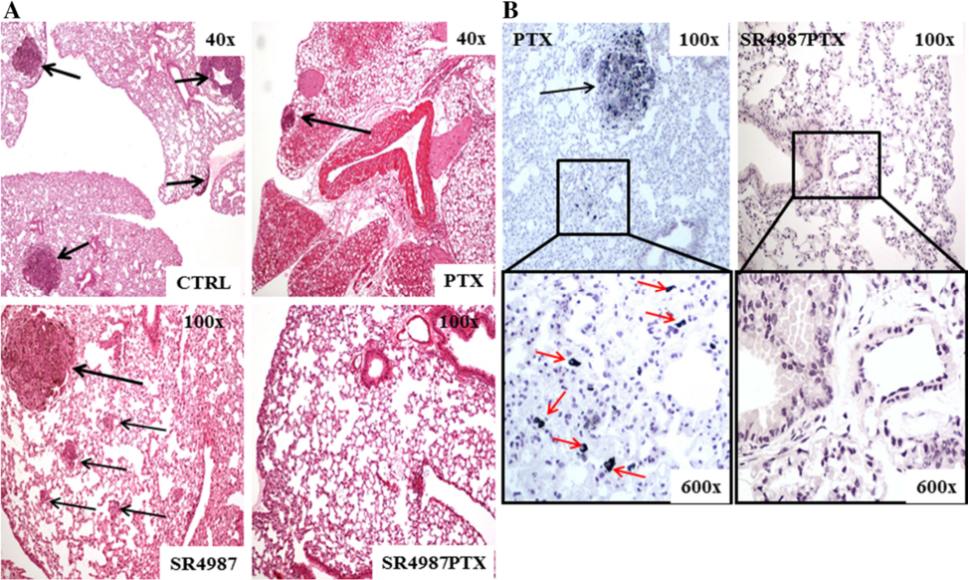
Histological examination of the lungs showing the potent anti-metastatic activity of SR4987PTX treatment. **a** HE staining shows the presence of several B16 lung nodules (black arrows) in control mice (CTRL) and in SR4987 treated mice. Some metastasis are present in mice treated with a very high dose of PTX (10 mg/kg), while mice treated with SR4987PTX appear without metastatic nodules. **b** Fontana-Masson staining shows the presence of lung micrometastasis (red arrows) in PTX and their absence in SR4987PTX treated mice (boxes 600x magnifications)

B16 melanoma cells were injected into the tail vein of syngeneic C57Bl6 mice at the dose of 2.5x10^5^/0.2 ml. On day 5 after tumor injection, mice were divided in 5 groups receiving respectively: saline (controls), PTX 10 mg/Kg, SR4987 at 1×10^5^/0.2 ml, SR4987PTX at 1×10^5^ /0.2 ml and at 5×10^4^/0.2 ml. Treatments were repeated on day 10 and 15 for a total of three administrations and all mice were sacrificed on day 21. As summarized in Table 1 (section A), SR4987PTX treatments produced a potent lung metastasis suppression at both doses used (around 90 % inhibition; *p* < 0.001 vs control). The number of B16 lung nodules was not affected by SR4987, while PTX treatments, given at the maximum tolerated dose (MTD) of 10 mg/kg, reduced metastasis (*p* < 0.01 vs control) but significantly less than SR4987PTX (*p* < 0.05). It is important to outline that the total dose of PTX given free was around 2,000 fold higher than mice that received PTX delivered by cells, thus highlighting the remarkable efficacy of SR4987PTX treatment. Repeating the experiment using SR4987PTX only at the low dose of 5×10^4^ we confirmed the potent anti-metastatic activity (Table 1 section B). Besides the quantification of lung metastasis performed under dissecting stereomicroscopy (Additional file 2: Figure S6), histology of the lungs confirmed that in control and in SR4987 treated mice several metastasis were present, whereas in mice treated with SR4987PTX, rare metastasis were seen (Fig. 1a). A similar picture was observed in mice treated with PTX alone (Fig. 1a). However, analyzing the lung parenchyma by Fontana-Masson staining, that lead to a better detection of micrometastasis, we observed that micrometastasis were practically absent in SR4987PTX treated mice. On the contrary in PTX treated mice some residual B16 micrometastasis were present (Fig. 1b). We conclude that three injection of PTX at MTD (10 mg/kg) was significant less effective than SR4978PTX that even had a superior activity in eliminating also micrometastasis

**Table 1:** SR4987PTX injected i.v. suppress B16 lung metastasis

Section A			
Groups	days of treatments	Median number of lung metastasis (interval)	% of Metastasis inhibition
Controls (saline)	0	37 (12-60)	-
PTX (10 mg/Kg)	5,10,15	11 (11-15)*	70.3
SR4987 (10^5^)	5,10,15	40 (16-51)	0
SR4987PTX (10^5^)	5,10,15	5 (2-9)**^,^°	86.5
SR4987PTX (5×10^4^)	5,10,15	3 (2-4)**^,^°	91.8°
Section B			
Controls (saline)	0	29 (12-32)	-
PTX (10 mg/Kg)	5,10,15	10 (9-11)*	65
SR4987PTX(5×10^4^)	5,10,15	3 (2-4)**^,^°	90°

Legend. On day 0, C57Bl6 mice were injected i.v. with 2.5×10^5^/0.2 ml B16 melanoma cells On day 5, 10 and 15 after B16 injection, mice were treated i.v. with saline (controls), PTX (10 mg/Kg), SR4987(10^5^/0.2 ml) and SR4987PTX at 10^5^ and 5×10^4^/0.2 ml respectively. On day 21 after tumor cells injection, mice were sacrificed and lung nodules counted under a dissecting stereomicroscopy. Numbers in the table are the median number and the range of metastases determined for each group of mice (12) and are the media of three different experiments. * *p* < 0.05; ** *p* <0.01 vs controls; **°** *p* < 0.05 vs PTX treated mice

.

### B16-CM and TNFα improve adhesion of SR4987PTX to microvascular endothelium and to stromal cells of the lung

**Fig. 2:**
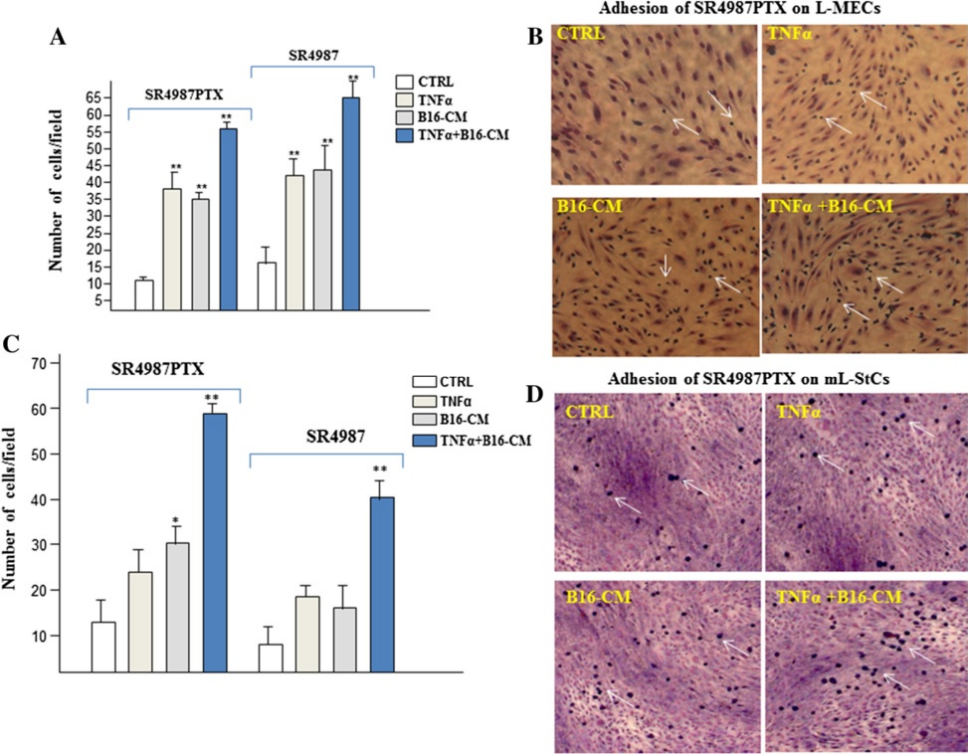
Adhesion of SR4987 and SR4987PTX to L-MECs and mL-StCs improved by B16-CM, TNFα and their association. **a** quantification of SR4987 and SR4987PTX that adhere to L-MECs monolayers not treated (CTRL) or treated with TNFα, B16-CM and TNFα + B16-CM respectively. **b** photographs (20x), showing the SR4987PTX cells (white arrows) that adhere on L-MEC monolayers. **c** quantification of SR4987 and SR4987PTX that adhere to mL-StCs monolayers. **d** photographs (20x) showing the adherent SR4987PTX cells on mL-StCs monolayer. The bars in the figure are the mean ± SD of cells counted with a calibrated eyepiece in 15 different fields at 40X magnification. Each test was run in quadruplicate. (*P < 0.05;**P < 0.01 vs CTRL)

In order to understand the mechanism of SR4987PTX homing to metastatic lung, we started to evaluate whether B16 cells could affect the capacity of SR4987PTX to interact to lung-derived microvascular endothelial cells (L-MECs) and/or to mouse lung-derived stromal cells (mL-.StCs). To this end, adhesion experiments were performed by seeding SR4987 and SR4987PTX on L-MECs and on mL-StCs monolayer. As shown in Fig. 2, both SR4987 and SR4987PTX had a poor capacity to adhere on L-MECs and mL-StCs, in contrast they adhere rapidly on L-MECs (Fig. 2a and b) and mL-StCs (Fig. 2c and d) after stimulation either with B16-CM or TNFα, an inflammatory cytokine that is known to increase adhesion molecules expression on both endothelium and StCs [30, 31]; to note that the combination B16-CM + TNFα significantly enhanced adhesion of SR4987 and SR4987PTX on both L-MECs and mL-StCs.

### Priming of mL-StCs with B16-CM alone or combined with TNFα stimulate SR4987PTX chemotaxis that is mediated by SDF-1/CXCR4/CXCR7 axis

**Fig. 3:**
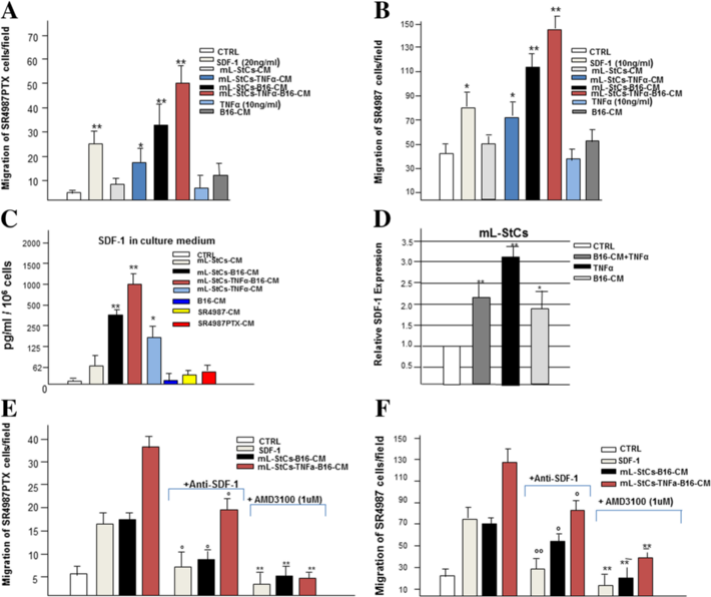
Chemotaxis of SR4987 and SR4987PTX induced by mL-StCs-CM upon priming with B16-CM or B16-CM + TNFα mediated by SDF-1/CXCR4/CXCR7 axis. In (**a**) SR4987PTX and (**b**) SR4987 cells migration. Bars are the mean ± SD of three independent experiments. Note the chemotactic activity of mL-StCs-B16-CM and mL-StCs-TNFα-B16-CM on both SR4987PTX and SR4987 (*P < 0.05;**P < 0.01 versus CTRL). B16-CM and mL-StCs-CM are not effective while SDF-1 induces SR4987PTX and SR4987 chemotaxis. In (**c**) SDF-1 secreted by the B16 melanoma cells and by mL-StCs under different stimuli. Bars are the mean value ± SD of SDF-1 detected in the CMs normalized for the same number of cells (10^6^). In (**d**) the analysis of SDF-1 at mRNA level in B16 and mLStCs under different culture conditions. Bars ± SD are the relative gene expression calculated by a comparative method (2^-ΔΔCt^) using GAPDH as an housekeeping gene. In (**e**) SR4987PTX and (**f**) SR4987 cells migration in the presence of anti-SDF-1 (1 μg/ml) or after cells priming with AMD3100 (1 μM). Note that both anti-SDF-1 and AMD3100 significantly reduce chemiotaxis (° < 0.05 versus no anti-SDF-1 addition; **p < 0.01 versus no AMD3100 treatment)

We next performed chemotaxis experiments by using trans-well insert. Under control medium (CTRL) condition, we found that SR4987PTX had a significant lower spontaneous motility than SR4987. Surprisingly, the addition of B16-CM, as chemotactic stimulus, did not substantially improve basic migration of both SR4987 and SR4987PTX (Additional file 2: Figure S7). We then tested the CM recovered from culture of mL-StCs (mL-StCs-CM) and again no significant increase of migration was observed. However, when mL-StCs were primed first with B16-CM (mL-StCs-B16-CM), TNFα (mL-StCs-TNFα-CM) or their combination (mL-StCs-TNFα-B16-CM), a potent induction of both SR4987 and SR4987PTX migration was noted (Fig. 3). The oriented movement of SR4987PTX was highest in the presence of mL-StCs-TNFα-B16-CM (around 10 fold higher than control) but either addition of mL-StCs-B16-CM and to lesser extent mL-StCs-TNFα-CM were effective (6 and 3 fold the CTRL respectively) (Fig. 3a). Untreated SR4987 behaved similarly to SR4987PTX (Fig. 3b). Additionally, we also found that SDF-1, a cytokine known to be a chemoattractant for MSCs [32] was effective to stimulate both SR4987PTX and SR4987 at 20 and 10 ng/ml respectively (Fig. 3a and b).

Since the efficacy of SDF-1 and because SR4987 cells expressed a high level of CXCR4 (90 %) and, to minor extent, CXCR7 (20 %) SDF-1 receptors [33] (Additional file 2: Figure S7), we then verified if SDF-1 could be a mediator of SR4987 and SR4987PTX chemotaxis in the metastatic lung. We first analyzed *in vitro* the capacity of B16 and mL-StCs to release SDF-1. We found that B16 cells and mL-StCs did not release a significant amount of SDF-1 in the CM (Fig. 3c). However, when mL-StCs were primed with B16-CM alone or combined with TNFα the concentration of SDF-1 in the medium was significantly increased (Fig. 3c). These results were also confirmed by analyzing the expression of SDF-1 by PCR performed on mL-StCs under the different treatments conditions (Fig. 3d).

Migration experiments of SR4987PTX and SR4987 were repeated by adding anti-SDF-1 antibodies or in the presence of AMD3100 a molecule able to bind specifically the SDF-1 receptors CXCR4 and CXCR7 [34]. Anti-SDF-1 antibodies and AMD3100 were both able to significantly reduce chemotaxis (Fig. 3e and f).

### Increment of SDF-1 expression and presence of SDF-1+ cells in the lung of mice bearing B16 melanoma metastasis

**Fig. 4:**
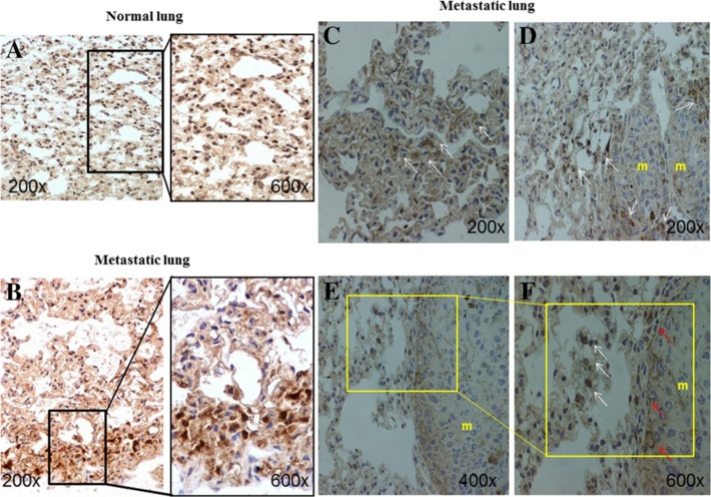
SDF-1 expression and presence of SDF-1+ cells in the lung of mice bearing B16-melanoma metastasis. **a**-**f** Immunostaining for SDF-1 of normal and metastatic lung. In (**a**) normal lung parenchyma shows no staining for SDF-1 at lower (200x left panel) and at higher magnification (600x right panel). In (**b**) metastatic lung parenchyma shows an intense SDF-1 staining at both magnifications (200x and 600x). In (**c**,**d**) the metastatic lung shows the presence of SDF-1+ cells (white arrows) in the parenchyma near or at periphery of metastasis (m). In (**e**,**f**) high magnification of a lung metastasis (m) showing the presence of SFD-1 + cells (white arrows) and a diffuse SDF-1 staining (red arrows) along the entire border of the metastasis

Subsequently, by using SDF-1 antibodies, we investigated the expression of SDF-1 and the localization of SDF-1+ cells in normal and in B16 metastatic lungs (Fig. 4). In summary SDF-1 expression and the SDF-1+ cells were practically absent in normal lung (Fig. 4a) but highly expressed in mice with metastasis (Fig. 4b). SDF-1+ cells were particularly localized in the lung stroma near or at periphery of metastasis, but absent inside metastatic nodules (Fig. 4c and d). Interestingly, the observation at higher magnification of the periphery of metastasis, revealed that SFD-1 staining was present in some cells but mostly widespread among cancer cells; while a diffuse staining along the extreme periphery of the metastases was noted (Fig. 4e and f).

### SR4987PTX cells localize preferentially in the vessels nearby metastatic nodule

**Fig. 5:**
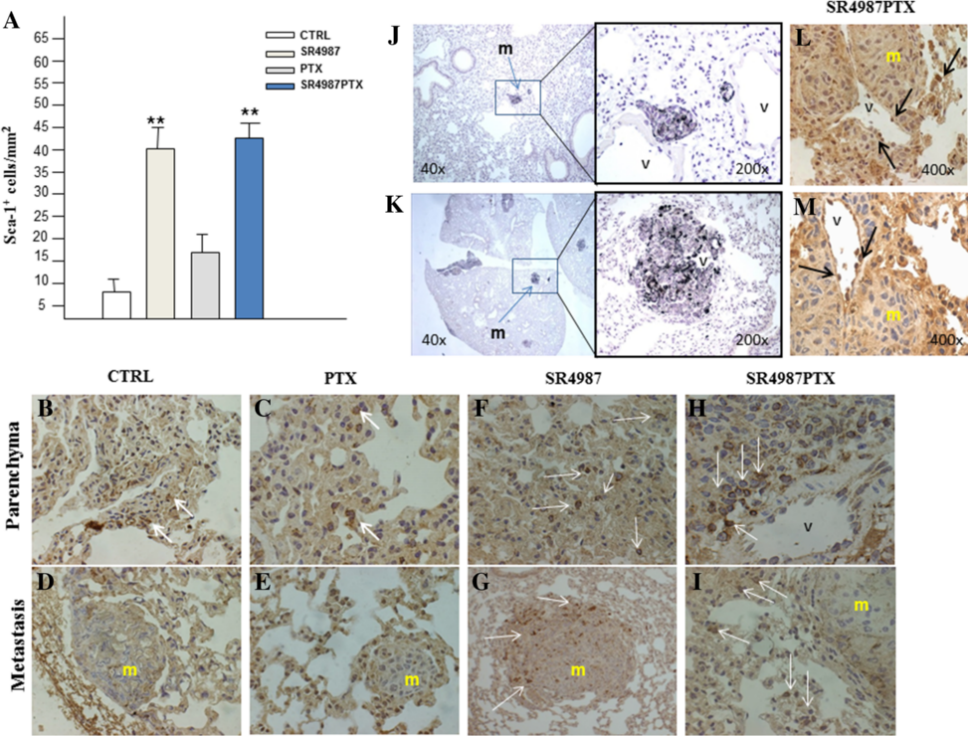
Sca-1+ cells in lung parenchyma of SR4987PTX injected mice localize in vessels nearby metastatic nodule. Anti-Sca-1 antibodies were used to investigate the capacity of SR4987 and SR4987PTX to home lung B16 nodules. **a** quantification of Sca-1 + cells in the lungs. Bars are the mean ± SD of three mice and are the number of Sca-1^+^cells/mm^2^ counted at 400X (**p < 0.01 vs CTRL or PTX treated mice). **b**,**c**,**f**,**h** Immunostaining for Sca-1 of metastatic lung parenchyma and (**d**,**e**,**g**,**i**) metastasis (m) area. Very few Sca-1+ cells (white arrows) are present in the lung of control and PTX treated mice in both parenchyma (**b**,**c**) and metastasis area (**d**,**e**). Mice treated either with SR4987 (**f**,**g**) and SR4987PTX (**h**,**i**) show many Sca-1+ cells in the lung parenchyma near the vessels (V) and at the periphery of metastatic nodules (200x magnifications). (J) Fontana-Masson staining showing B16 metastasis nearby (top left panel) or surrounding a lung microvessel (bottom right panel). In (L,M) Sca-1+ cells (black arrows) are attached to endothelium of tumor infiltrating microvessels

To detect the presence and to roughly quantify the number of SR4987PTX in the metastatic lung, anti-Sca-1 antibodies were used (Fig. 5). We found that mice treated with either SR4987 and SR4987PTX had almost 5-6 fold Sca-1+ cells/mm^2^ higher than control and 3 fold of PTX treated mice (Fig. 5a). The presence of few Sca-1+ cells in control and PTX treated mice suggested a little endogenous mobilization of MSCs from BM (Fig. 5b and c). However, in these mice, Sca-1+ cells were not seen nearby metastasis (Fig. 5d and e). In mice treated with SR4987, we found several Sca-1+ cells in lung parenchyma (Fig. 5f), but even within metastases (Fig. 5g). Finally in mice treated with SR4987PTX a significant presence of Sca-1+ cells in close proximity of microvessels (Fig. 5h) and nearby metastatic nodule were observed (Fig. 5i). Substantially, Sca-1+ cells were detected near the microvessels where most of the metastasis were localized (Fig. 5j). By a more accurate observation, we discovered that some Sca-1+ cells were even present in the lumen of microvessels infiltrating metastatic nodule (Fig. 5i and m), demonstrating that SR4987PTX cells could reach metastasis through the hematic route and that they may strongly affect cancer cells growth even if they do not cross the vessels wall.

## Discussion

MSCs may represent a novel therapeutic tool for fighting cancer [1]. MSCs can be engineered to produce and delivery anti-cancer molecules [3–6, 35, 36] or, as our group have recently demonstrated, MSCs can become Dr-MCs upon priming with anti-cancer drug like PTX, acquiring strong anti-cancer activity both and *in vivo* [15, 16]. Paclitaxel is an important molecule active on many solid tumors including melanoma [37] and also with anti-angiogenic activity. PTX priming of MSCs induces mitotic arrest of the cells without affecting their viability and allowing to the uptake/release mechanism to properly works. These features have been verified also for Vincristine and drugs with other mechanism of action as Gemcitabine and Doxorubicin. In any case, regardless the kind of manipulation used, it is necessary that MSCs maintain their homing ability because is crucial to localize their cytotoxic cargo in the tumor. At this regard, the therapeutic localization of MSCs in organs such as lung is certainly facilitated by i.v. administration [10, 11].

To expand our previous work, we here investigated whether the i.v. injection of Dr-MCs loaded with PTX may inhibit the formation of lung metastases, induced in C57Bl6 mice upon i.v. injection of syngeneic B16 melanoma cells. To exclude any immunological interference by the host, as Dr-MCs, we used the SR4987 cells, a BM-derived MSCs line syngeneic to C57Bl6 mice [23] and able to uptake and release PTX and to kill cancer cells [15, 16].

We found that 5×10^4^ SR4987PTX cells injected i.v. three times, starting from day 5 after tumor injection, were sufficient to produce up to 90 % of lung metastasis inhibition; an effect that was even superior of those obtained with PTX given at the MTD of 10 mg/Kg. Because SR4987 may incorporate at maximum around 2.5 pg/cell of PTX [15], the dose of PTX that mice received through the cells was about 2000 times less than the groups treated with PTX. Thus, we conclude that the delivery of the drug through the cells has a significantly higher therapeutic index than the PTX given through the standard therapy. Previous reports have shown the efficacy of engineered MSCs delivering anti-cancer molecules, or MSCs per se, to inhibit lung metastases upon i.v. administration [5, 6, 38–40]. Our results expand these observations, and demonstrate for the first time that syngeneic MSCs loaded with PTX given systemically in mice bearing lung metastasis, have a potent therapeutic effect.

The capacity of SR4987PTX to home malignant lung nodules was investigated both *in vitro* and *in vivo*. In summary our results indicate that SR4987PTX once injected i.v., are able to reach metastatic lung nodules through a complex mechanism involving mainly the chemokine SDF-1/CXCL12 and its receptors CXCR4/CXCR7. These data confirm previous reports showing the importance of SDF-1/CXCR4/CXCR7 axis in the homing of MSCs to cancer environment [41–44]. However, besides this, our study highlights a new undiscovered mechanism of MSCs homing in metastatic lung that is not mediated directly by B16 cells but through the implication of lung StCs. Indeed, our *in vitro* data show that B16-CM neither stimulates chemotaxis of both SR4987 and SR4987PTX nor contains significant amount of SDF-1. In contrast, CM derived from mL-StCs primed with B16-CM, secretes SDF-1 and stimulates chemotaxis of SR4987PTX. Further, the *in vivo* analysis of the SDF-1+ cells localization in metastatic lung, reveals that most of the SDF-1+ cells are not present inside metastasis but are localized in the lung stroma or at the extreme periphery of metastatic nodules. We therefore conclude that SR4987PTX, once arrested in the lung, migrate toward B16 metastasis by an “indirect mechanism” mediated by SDF-1 secreted by StCs of the lung under the influence of factors released by B16 cancer cells. In this contest, TNFα seems to play an important role as enhancer of the B16 cancer cells activity.

Our results thus highlight the importance of StCs surrounding the metastatic nodule that seem to behave like tumor stromal cells in favoring the progression of primary cancer [45–47]. Whether this StCs-mediated attraction of MSCs is occurring in other metastatic organs besides lung, remains to be established. The finding that lung contains specific resident MSCs that differ from those from BM [48], may suggest an organ-specific activity of lung MSCs in controlling local tissue diseases [49, 50].

Finally, the *in vivo* search of SR4987PTX in metastatic lung demonstrated the presence of many Sca-1+ cells in parenchyma and even inside metastatic nodules. The few Sca-1+ cells present in the lung of control and PTX treated mice suggested a modest endogenous mobilization of MSCs from BM, at least at the time of mice sacrifice. Notably, we observed that Sca-1+ cells in mice injected with SR4987PTX were mostly detected nearby metastases or even attached to endothelium of the microvessels infiltrating metastatic foci; thus very close to cancer cells. To note that the vicinity of Dr-MCs to cancer cells is an important condition to ensure the efficacy of their toxic cargo (PTX) [15, 16]. In our opinion this result could explain the potent anti-metastatic effect obtained even when mice were treated with a lower dose of SR4987PTX.

## Conclusions

Tumor recruits MSCs from host BM to form its stroma [41, 42, 51] a mechanism probably essential for tumor progression [45–48]. We can use this cancer property to fight it by administrating exogenous MSCs that can work as Trojan horses carrying in the tumor anticancer molecules [1]. Although the presence of some conflicting data [52, 53], in general preclinical studies indicate that MSCs loaded with anti-cancer molecules could be very effective to inhibit primary and metastatic cancer growth [3–6]. The results shown here clearly demonstrated that our approach to upload anticancer drug, such as PTX, on MSCs lead them to become strongly cytotoxic against cancer and, importantly, without altering their home properties. Actually, a cure for lung metastasis in humans is mostly unlikely and we do not know whether a therapy combining engineered MSCs and Dr-MCs may work synergistically. However, we think that our approach using Dr-MCs loaded with PTX may represent a new valid and additive therapeutic tool to fight lung metastases and, perhaps, primary lung cancers in human.

## Additional files

Additional file 1: **Supplementary materials and methods.** (DOC 45 kb)

Additional file 2: Figure S1. SR4987GFP and SR4987GFP-PTX arrest in the lung of mice upon i.v. administration. Mice were sacrificed at 6 h, 24 h and 48 h after treatments with cells. Panel (A) shows photos taken under fluorescent microscopy immediately upon enzymatic lung tissue disruption, showing the presence of several fluorescent cells until 48 h after their injection. Not significant difference were noted between mice treated with SR4987GFP and SR4987GFP-PTX (10x magnification). Panel (B) shows photos of SR4987GFP and SR4987GFP-PTX cultured for 2 and 7 days upon isolation from mice, indicating that cells maintain their viability (20x magnification). **Figure S2.** Sca-1 is highly expressed on SR4987 and can be used for their detection in the mice lung. In (A) FC analysis showing the high positivity of the SR4987 for Sca-1. In (B), Immunostaining for anti-Sca-1 of lungs derived from mice treated i.v. with saline , 10^6^ SR4987 and 10^6^ SR4987PTX cells and sacrificed 24 h after treatments. Pictures show the high presence of Sca-1+ cells (black arrows) in the parenchyma and in the lumen of lung vessels in mice treated with both SR4987 and SR4987PTX. Only, very few Sca-1+ cells were detected in control mice (CTRL) (photo 20x, in the box 40x magnifications). **Figure S3.** Sensibility of SR4987 to PTX and efficacy of SR4987PTX-CM to inhibit B16 proliferation. In (A) the proliferation of SR4987 in the presence of increasing concentration of PTX. The IC50 of PTX was around 34 ng/ml. In (B) is shown that viability of SR4987 is not affected by PTX even at concentration up 5000 ng/ml. In (C) is shown the growth inhibition of Molt-4 tumor cells in the presence of decreasing dilution of SR4987PTX-CM confirming that SR4987PTX release significant amount of PTX in the CM. In (D) the inhibition of proliferation of B16 cells in the presence of increasing concentration of PTX. The IC50 is 7.18 ± 4.99 ng/ml. In (E) is shown the dose dependent efficacy of SR4987PTX-CM to inhibit B16 cells proliferation with an IC50 DIL of around 1/10. **Figure S4.** Co-culture of SR4987 and SR4987PTX with B16 cells using transwell insert. B16 cells seeded on the top of membrane (0.4 um pore size) insert were co-cultured with different dose of SR4987 and SR4987PTX seeded on the bottom well and cultured for 5 days. The Figure shows that SR4987PTX at 1:5 ratio was able to strongly inhibit B16 proliferation as visualized by crystal violet staining of B16 in the transwell line (A) and on the membrane line (B). In line (C), photos of cultured B16 cells not stained upon co-culture or not with SR4987 and SR4987PTX (20x magnifications). **Figure S5.** Rosette formed by B16 + SR4987 and B16 + SR4987PTX and their ultrastructural analysis by TEM. In (A) and (B) rosette formed by SR4987GFP + B16 photographed under light and fluorescence microscopy respectively. (C-F) four different panels at TEM showing the interactions between untreated SR4987GFP and B16 cells. Note that B16 cells bound SR4987GFP appear healthy (C); the presence of area of contact between B16 and SR4987 is evident; junctional-like structure and a gap-like junction (D,E) and even nanotubule structure (F) are seen (white arrows). In (G) and (H) rosette formed by SR4987GFP-PTX + B16. Note that most of B16 cells appeared damaged and necrotic under light (G) and fluorescence (H) microscopy observation. In (I-L) four different panels at TEM showing the interaction between SR4987GFP-PTX and B16. The B16 cells bound to SR4987GFP-PTX show varying degrees of degeneration, some cells seemed even emptied of their cytoplasm (I) and, similarly to untreated SR4987, area of contact (white arrows) can be observed (J,K). In (L) a microvescicole that arise from SR4987PTX is photographed. **Figure S6.** Metastasis in the lung of control mice and after treatment with PTX, SR4987 and SR4987PTX. On day 21 after B16 cells injection, mice were euthanized, lungs removed and fixed in Bouin’s solution. In the figure photos of the lungs, taken under a dissecting stereomicroscope. Note the presence of several melanoma lung nodules (black arrows) in control and SR4987 treated mice. In contrast, at both dosage of SR4987PTX injected, the lung nodules are very few and are significantly less than those detected in PTX treated mice. **Figure S7.** Chemiotaxis of SR4987 and SR4987PTX in response to B16-CM and expression of the SDF-1 receptors CXCR4 and CXCR7*.* In (A) migration of SR4987PTX and SR4987 placed on the top of transwell membrane insert and stimulated by different dilution of B16-CM. No significant chemotaxis activity of both SR4987 and SR4987PTX is stimulated by B16-CM at every dilutions tested. Note that the basal migration of SR4987PTX is around three fold lower than untreated SR4987 (* *p* < 0.05). In (B) is showed FC analysis of SR4987 for the expression of the SDF-1 receptors CXCR4 and CXCR7, indicating the high positivity of the cells particularly for CXCR4 (>90 %), CXCR7 is less expressed (18 %). (DOC 26 kb)
