# Supplementary material for: Drug-releasing mesenchymal cells strongly suppress B16 lung metastasis in a syngeneic murine model
Source: J Exp Clin Cancer Res. 2015 Aug 13;34(1):82. doi: 10.1186/s13046-015-0200-3 (PMC4534150; doi:10.1186/s13046-015-0200-3)
Supplement: Supplementary file 1 — Supplementary materials and methods. (DOC 45 kb) [file 13046_2015_200_MOESM1_ESM.doc]

# Supplementary materials and methods

**Drug**

Paclitaxel (PTX) was purchased from Vinci-Biochem (Italy). The stock solution was prepared in DMSO (Sigma-Aldrich, USA) at a concentration of 5 mg/ml. Aliquots were stored at −20 °C. Working solutions were prepared fresh according to the experimental design by serial dilution in culture medium.

**Cells**

The murine cell line B16 (Mouse melanoma, melanin producing) (21) and Molt-4 (human acute T-lymphoblastic leukemia) (22) were provided by Centro Substrati Cellulari, ISZLER (Brescia, Italy). B16 cells were maintained in RPMI 1640 medium (Euroclone, UK) supplemented with 10% fetal bovine serum (FBS) (LONZA Walkersville MD USA) by weekly 1:5, 1:10 passages. Cells from passages 60 to 80 were used. Molt-4 cells were cultured in complete IMDM +10% FCS (Lonza) and weekly passages at ratio 1:20. The Mesenchymal stromal cell line SR4987 was established from a long-term BM-derived cell culture of BDF/1 mice (23) and is currently available at ATCC (CRL-2028). SR4987 cells were cultured in IMDM supplemented with 5% FBS and 2 mM L-glutamine (EuroClone, UK); and were weekly passed by 0.05% trypsin/0.02% EDTA (EuroClone, UK) at ratio 1:10 to 1:20 in T25 flasks (Corning, USA). In our experiments, cells from passages 120 to 140 were used. SR4987 are positive for stem cell marker Vimentin, CD44+, CD73+, CD105+, CD106+, Sca-1+, CD34+, and CD45+ and have the capacity to differentiate into osteoblasts and chondrocytes, but unable to differentiate into mature adipocytes (23). To study the arrest SR4987 in the lung upon i.v. injection, cells were transduced with GFP using lentiviral vector ( pCCLsin.PPThPGK.GFPpre) as previously described (15).

Human lung-derived microvascular endothelial cells (L-MECs) were obtained from Dr. Arnaldo Caruso (Laboratory of Microbiology, University of Brescia, Brescia, Italy). L-MECs were cultured in endothelial basal growth medium (EBM-GM) consisting of EBM medium supplemented with 5% FCS and EC mitogens (EGM bullet kit, LONZA Walkersville MD USA). L-MECs were routinely maintained in EBM-GM on Collagen+Fibronectin coated T25 flasks and passed once a week at 1:2 splitting dilution. Results presented in this study were conducted with L-MECs harvested upon 3 to 5 *in vitro* passages.

Mouse lung stromal cells (mL-StCs) were freshly isolated from C57Bl6 mice. Briefly, lungs were finely minced with scissor and then digested for 2h at 37°C in IMDM containing 0.25% collagenase D( Boehringer Mannheim Germany) and 0.2% Bovine Serum Albumin (BSA). The cell suspension was washed with PBS (Euroclone Celbio Milan, Italy) passed through a 40 μm pore size filter and then plated onto Petri dishes in IMDM +10%FBS. Cells were incubated and left to adhere overnight at 37°C, 5% CO_2_ while the floating cells were aspirated and discarded. The adherent cells were cultured IMDM +5% FBS. One week later, the primary cultures were detached by tripsinization and analyzed for mesenchymal markers CD44, CD29 and CD90 ( all purchased from Abcam Cambridge UK, mouse mesenchymal stromal cells panel) to confirm their mesenchymal origin. The mL-StCs cultures were maintained in IMDM+5% FBS and passed once a week at 1:2 splitting dilution. Results presented in this study were conducted with mL-StCs at 3 to 6 *in vitro* passages.

**PTX priming of SR4987 cells**

The toxic activity of PTX on SR4987 cells was determined in a 24h MTT (3-(4,5-dimethyl-2-thiazolyl)-2,5-diphenyl-2-Htetrazoliumbromide) assay (cytotoxicity test) and in a 7 day MTT assay (anti-proliferative test) as previously described (24). Based on these results, priming of SR4987 with PTX (SR4987PTX) was carried out with high drug dosage according to the procedure previously described (15). Briefly, subconfluent cultures (3×10^5^) of SR4987 cells were exposed to 2,000 ng/ml PTX. After 24h of incubation, the cultures were washed twice with PBS, trypsinized and washed twice in HBSS. Cells were then seeded in a new flask. After 24h of culture, the conditioned medium (CM) was collected and tested for antiproliferative activity *in vitro* on Molt-4 cells, a PTX sensitive cell line (IC50 = 7.18 ± 4.99 ng/ml). CM from untreated SR4987 cells cultured in the same conditions was used as control.

**Antitumor *in vitro* assay**

The effect of CM from control and SR4987PTX cells on B16 cells proliferation was studied in 96 well plates (Sarstedt, Germany) according to a MTT (3-(4,5-dimethyl-2-thiazolyl)-2,5- diphenyl-2-H-tetrazoliumbromide) assay (24). The inhibitory concentrations (IC_50_ and IC_90_) were determined according to the Reed and Muench formula (25). The antitumor activity of CM from SR4987PTX cells was compared to that of pure PTX and expressed as PTX equivalent concentration (PEC) according to the following algorithm: PEC (ng/ml) = DF_50_CM × IC_50_PTX (DF_50_CM is the dilution factors (DF) at which CM from SR4987PTX produced 50% growth inhibition; IC_50_PTX is the concentration (ng/ml) of PTX producing 50% of inhibition. The PTX released by a single cells was calculated dividing PEC for the number of cells seeded: and expressed as pg/cell.

To establish the optimal dose of SR4987PTX to inject , co-cultures with B16 cells were made by using transwell inserts (Becton Dickinson, USA). SR4987 and SR4987PTX were seeded, at different concentrations in wells of a 24 multiwells plate in IMDM+5%FCS. Into each transwell insert (0.4μm pore size) around 10^3^ B16 cells were plated in 200 μL medium. Five days post incubation (37°C, 5% CO_2_) medium from inserts was removed, B16 cells growing on the insert were washed with PBS 1X, 0.25% crystal violet (Fluka, USA) was added and incubated for 10’. The dye was removed, rinsed with tap water and allowed to air dry. When completely dried, 33% glacial acetic acid (500 µl/insert) was added to each insert. The eluted dye was taken in triplicate in 96 well plates (Sarstedt, Germany) and adsorbance was measured at 550 nm with a 96 well-plate reader (ChroMate, Awareness Technology, USA). Controls consisted in B16 cells cultured in the absence of SR4987 and cultured upon addition of PTX at 10ng/ml in the well.

**Rosette adherence assay and Transmission electron microscopy (TEM) analysis**

To study the interaction between B16 cells and SR4987, a rosette test was performed (16). Briefly, B16 and SR4987cells, loaded or not with PTX, were mixed in a conical tube in 500 µl of IMDM + 5% FBS (ratio B16/SR4987 5:1). After 24h of incubation at 37°C in air + 5% CO_2_ without stirring, 20 µl of cells were collected by a micropipette by aspirating the pellet lying on the tube bottom, and then transferred on a slide to evaluate rosette formation under an inverted microscope. Rosette from co-cultures of B16 and SR4987 primed or not with PTX were analyzed by TEM. Briefly, rosette were washed with 0.1 M pH 7.2 cacodylate buffer (CB) and then fixed in 2.5% glutaraldehyde in CB for 1h at room temperature. After washing in CB, the cell pellets were post-fixed in Osmium tetroxide (1% in CB) for 1h at room temperature, dehydrated in graded ethanol up to absolute, cleared in propylene oxide, preinfiltrated and embedded in epoxy resin (Epon 812). Hardened resin blocks were trimmed and sectioned on a ultramicrotome (Reichert Jung Ultracut). For light microscopic preliminary observation, 1 µm thick sections were stained with toluidine blue and observed under a light microscope (Nikon Eclipse E800). Ultrathin sections (90 nm) were mounted on 200-mesh copper grids, stained with uranyl acetate for 30’, and examined in a Philips EM 208 transmission electron microscope equipped with a DXM 1200 digital camera (CUME - Laboratory of Electron Microscopy, University of Perugia).

**Adhesion assay (Bolt)**

Adhesion of SR4987 and SR4987PTX to L-MECs were performed following a previously described procedure (16). Briefly, L-MECs were plated onto collagen-coated 24-multiwell plates at a concentration of 5x10^4^/500 µl in EBM-GM. The plates were incubated for 4 to 5 days to obtain a cell monolayer. L-MECs monolayer was stimulated by adding B16 derived CM (B16-CM) recovered from 72h culture, tumor necrosis factor-alpha (TNFα; 10 ng/ml; Sigma Chemical) and their combination for 24h. L-MECs were then washed with PBS and allowed to interact with SR4987 and SR4987PTX (10^4^ cells/well) for 1h in IMDM +0.2%BSA. The unbound SR4987 and SR4987PTX were removed by three washes with warm PBS while the attached cells to L-MECs were fixed for 5’ with 100µl of cold methanol and stained with Diff Quick (Merz-Dade, Dudingen, Switzerland) at room temperature. The plates were then washed several times with deionised water and the cells bound to L-MECs were counted with a calibrated eyepiece in 15 different fields at 40× magnification. Each test was run in quadruplicate. Adhesion of SR4987 and SR4987PTX to confluent mL-StCs was carried out in same manner to that described for L-MECs

**Migration assay**

Transwell supports were used to test spontaneous migration and chemotaxis of SR4987 and SR4987PTX. Migration assay was performed as previously described (**26**). Briefly, 6.5 mm Transwell 5 μm pore size Polycarbonate membrane insert were coated with Collagen type-1 and for each test 10^5^ cells in 200 μl of IMDM+0.2% BSA were routinely placed on the top of the membrane insert ( the upper compartment of the well). Test samples were placed in the lower compartment (a substance placed in the lower compartment of the well acts as chemoattractant and the cells move from the surface through the membrane against a concentration gradient). To evaluate spontaneous migration 500 μl of control IMDM+0.2% BSA medium were used. To evaluate SR4987 and SR4987PTX chemotaxis, in the lower compartment we tested mouse SDF-1a (1-100 ng/ml) (R&D system), TNFα (1-50ng/ml), B16-CM (1:1-1:100 dilutions) mL-StCs-CM (1:1-1:100 dilutions). Additionally In the lower compartments we also placed CM derived from mL-StCs primed for 24h either with B16-CM (1:1) ( mL-StCs-B16-CM), TNFα (10ng/ml) (mL-StCs-TNFα-CM) or B16-CM+TNFα (mL-StCs-B16-TNFα-CM). The blocking effect of monoclonal antibodies (mAbs) anti mouse SDF-1 (R & D System), as well control mouse IgG, (0.01-1ug/ml) was tested by mixing antibodies in the lower compartment with SDF-1 or with CMs. To block SDF-1 receptors CXCR4/CXCR7, SR4987 and SR4987PTX (5x10^5^) were pre-incubated for 1h at 37°C in IMDM+0.2% BSA in presence of different concentration of AMD3100 (0.1-10 µM) (purchased from Sigma). Migration assay was carried out for 6h at 37°C in 5% CO_2_. At the end of incubation, the membrane inserts were fixed with methanol and stained with Diff Quick. Cells attached to the upper surface of the membrane were removed with a swab and the cells migrated across the membrane were counted in the lower surface. Data reported represent media ± standard deviation (SD) of the cells counted in 10 different fields for each membrane at 400 x magnification. Each determination was done in duplicate.

**Immunoassay for mouse SDF-1**

CM samples recovered from 48h cultured SR4987, SR4987PTX, B16 cells, or from mL-StCs stimulated or not with B16-CM, TNFα and B16-CM+TNFα were analyzed for the presence of SDF-1 using Elisa kit (R & D System). The value of SDF-1 detected in the CM were normalized for the same number of cells counted at the end of incubation.

**Flow Cytometry**

To dertermine the expression of Sca-1, CXCR4 and CXCR7 on SR4987 Flow cytometry (FC) was used. Briefly, SR4987 cells were, trypsinized, washed and resupsended with FC buffer (PBS BSA 0.5%, sodium azide 0.02%, pH 7.2) and fixed with paraformaldehyde 2%. Cells (1x10^5^/500 µl) were incubated for 20’at room temperature, in the dark, with anti-Sca-1 FITC, anti-CXCR4 (ab1670) and anti-CXCR7 antibodies (all purchased from Abcam Cambridge UK) and then washed with FC buffer. 20,000 events were acquired for each analysis using Epics “XL-MLC” (Beckman Coulter,USA) and histogram elaboration was performed with EXPO 32 software.

**RT-PCR Quantitative Reverse Transcriptase–Polymerase Chain Reaction**

For the analysis of mRNA levels 1000 ng of total RNA isolated using the RNeasy kit (Qiagen) was reverse-transcribed using iScript cDNA Synthesis Kit (Bio-Rad Laboratories). Triplicate polymerase chain reactions were carried out on an CFX 96 Touch Real Time PCR Detection System (Bio-Rad Laboratories). Relative gene expression was calculated by a comparative method (2^-ΔΔCt^) using GAPDH as an housekeeping gene. Primers sequences were designed using Primer3 software.

***In vivo* experiments.**

Six-to 8-week old male C57BL/6 mice were purchased from Charles River (Calco, Milan, Italy). All the animal experiments were performed at the Animal Facility of Regina Elena National Cancer Institute in Rome, Italy. The procedures involving mice and care were in compliance with our institutional animal care guidelines and with international directives (directive 2010/63/EU of the European parliament and of the council; Guide for the Care and Use of Laboratory Animals, United States National Research Council, 2011). Preliminary experiments were performed to confirm the capacity of SR4987 and SR4987PTX to arrest in the lung upon i.v. injection. At this end, healthy mice (2 each group) were treated i.v. with saline, 10^6^ SR4987GFP and 10^6^ SR4987GFP-PTX cells. Mice were then sacrificed at 6h, 24h and 48h after treatments. Lungs were dissected, minced and digested by collagenase (for 2h at 37°C) the obtain a lung cell suspension that was rapidly analyzed under Fluorescent microscopy to detect the presence of SR4987GFP and SR4987GFP-PTX cells. A second serie of mice ( 2 each group) were treated with PTX, SR4987 and SR4987PTX, lung were removed after 24h and fixed in 5% formalin. F**ormalin-fixed lungs were dehydrated and embedded in paraffin, serial 4-**μm-thick paraffin sections were processed for immunohistochemistry (**27**) for the detection of SCa-1+cells by using rabbit monoclonal anti-Sca1/Ly6A/E antibody (1:150, Abcam).

To study the anti-metastatic activity of SR4987PTX, mice were injected i.v. with B16 melanoma cells at 2.5 x 10^5^ cells/0.2ml. On day 5, after B16 injection, mice were divided in 5 groups and treated i.v. with 0.2ml saline (controls), PTX given in free formulation at 10mg/Kg, SR4987(10^5^/0.2ml) , SR4987PTX at 10^5^ and 5x10^4^/0.2ml respectively. Treatments were repeated on day 10 and 15 for a total of three injections. On day 21 after tumor cells injection, mice were euthanized, lungs removed and fixed in Bouin’s solution. The number of lung nodules were counted and photographed with the professional digital compact camera (Olympus) under a dissecting stereomicroscope. Each group included twelve mice.

To investigate the capacity of SR4987 and SR4987PTX to home lung B16 nodules, mice (3 each group) were injected i.v. with 10^6^ B16 cells, upon 14 days mice were i.v. treated with saline (controls), PTX (10mg/kg), SR4987 (10^5^) and SR4987PTX (10^5^) and sacrificed 48h after treatments. The lungs were removed fixed in formalin, embedded in paraffin and processed by immunohistochemistry , as described above, for Sca-1+cells detection. The number of Sca-1^+^cells was evaluated at high power field (HPF), 400X magnification. At least 10 fields for each case were randomly evaluated with variability less than 5%.

**Histology and Immunohistochemical Studies**

Lungs from control and treated mice were fixed in 10% of formalin, embedded in paraffin and then processed for histology and immunohistochemistry. Hematoxylin and eosin (HE) stained serial sections of five micrometer thickness were used for histological evaluation of lung metastasis. Lung metastasis and micrometastasis were also investigated by Fontana-Masson staining. Briefly, deparaffinized and rehydrated sections were incubated in Lugol solution for 10 minutes. After washing in distilled water, the slides were incubated in Fontana silver nitrate working solution for 16-18 hours, in a Coplin jar protected from light. After washing in distilled water, the  slides were incubated in 5%  sodium thiosulfate (1-2 minutes), rinsed in distilled water, counterstained with nuclear red solution for 10 minutes, rinsed in distilled water again, dehydrated  in the ethanol  series (70, 96 and 100 %, v/v), cleared in xylene and mounted with Eukitt®. Lungs were immunostained for Sca-1 and SDF-1 expression according to standard protocols (**26**). Briefly, lung sections were transferred to glass slides coated with poly-lysine, deparaffinized in 100% xylene, and rehydrated in graded ethanol. After heat-induced antigen retrieval, endogenous peroxidase was inhibited by incubating tissue sections with 3% hydrogen peroxidase for 15’ at room temperature, while aspecific epitope binding was blocked by incubation for 20’ with 20% human serum. All samples were then processed by the avidine-biotin peroxidase complex method according to manufacturer’s recommendations (LASB kit DakoCytomation, Carpenteria CA, USA). Rabbit monoclonal anti-Sca1/Ly6A/E antibodies (1:150) and Polyclonal antibodies against human and mouse SDF-1 (1:100-500) (Santa Cruz Biotechnology Inc USA) were used to detect the presence of SR4987 and SR4987PTX and to investigate the expression of SDF-1+ in the lungs respectively.
