# Supplementary material for: Drug-releasing mesenchymal cells strongly suppress B16 lung metastasis in a syngeneic murine model
Source: J Exp Clin Cancer Res. 2015 Aug 13;34(1):82. doi: 10.1186/s13046-015-0200-3 (PMC4534150; doi:10.1186/s13046-015-0200-3)
Supplement: Supplementary file 2 — SR4987GFP and SR4987GFP-PTX arrest in the lung of mice upon i.v. administration. Mice were sacrificed at 6 h, 24 h and 48 h after treatments with cells. Panel (A) shows photos taken under fluorescent microscopy immediately upon enzymatic lung tissue disruption, showing the presence of several fluorescent cells until 48 h after their injection. Not significant difference were noted between mice treated with SR4987GFP and SR4987GFP-PTX (10x magnification). Panel (B) shows photos of SR4987GFP and SR4987GFP-PTX cultured for 2 and 7 days upon isolation from mice, indicating that cells maintain their viability (20x magnification). Figure S2. Sca-1 is highly expressed on SR4987 and can be used for their detection in the mice lung. In (A) FC analysis showing the high positivity of the SR4987 for Sca-1. In (B), Immunostaining for anti-Sca-1 of lungs derived from mice treated i.v. with saline , 106 SR4987 and 106 SR4987PTX cells and sacrificed 24 h after treatments. Pictures show the high presence of Sca-1+ cells (black arrows) in the parenchyma and in the lumen of lung vessels in mice treated with both SR4987 and SR4987PTX. Only, very few Sca-1+ cells were detected in control mice (CTRL) (photo 20x, in the box 40x magnifications). Figure S3. Sensibility of SR4987 to PTX and efficacy of SR4987PTX-CM to inhibit B16 proliferation. In (A) the proliferation of SR4987 in the presence of increasing concentration of PTX. The IC50 of PTX was around 34 ng/ml. In (B) is shown that viability of SR4987 is not affected by PTX even at concentration up 5000 ng/ml. In (C) is shown the growth inhibition of Molt-4 tumor cells in the presence of decreasing dilution of SR4987PTX-CM confirming that SR4987PTX release significant amount of PTX in the CM. In (D) the inhibition of proliferation of B16 cells in the presence of increasing concentration of PTX. The IC50 is 7.18 ± 4.99 ng/ml. In (E) is shown the dose dependent efficacy of SR4987PTX-CM to inhibit B16 cells proliferation with an IC50 DIL o [file 13046_2015_200_MOESM2_ESM.doc]

# Supplementary Figure legends

# Supplementary Figure 1

# SR4987GFP and SR4987GFP-PTX arrest in the lung of mice upon i.v. administration. Mice were sacrificed at 6h, 24h and 48h after treatments with cells. Panel (A) shows photos taken under fluorescent microscopy immediately upon enzymatic lung tissue disruption, showing the presence of several fluorescent cells until 48h after their injection. Not significant differences were noted between mice treated with SR4987GFP and SR4987GFP-PTX (10x magnification). Panel (B) shows photos of SR4987GFP and SR4987GFP-PTX cultured for 2 and 7 days upon isolation from mice, indicating that cells maintain their viability (20x magnification)

# Supplementary Figure 2

# Sca-1 is highly expressed on SR4987 and can be used for their detection in the mice lung. In (A) FC analysis showing the high positivity of the SR4987 for Sca-1. In (B), Immunostaining for anti-Sca-1 of lungs derived from mice treated i.v. with saline , 10^6^ SR4987 and 10^6^ SR4987PTX cells and sacrificed 24h after treatments. Pictures show the high presence of Sca-1+ cells (black arrows) in the parenchyma and in the lumen of lung vessels in mice treated with both SR4987 and SR4987PTX. Only, very few Sca-1+ cells were detected in control mice (CTRL) (photo 20x, in the box 40x magnifications)

**Supplementary Figure 3**

**Sensibility of SR4987 to PTX and efficacy of SR4987PTX-CM to inhibit B16 proliferation.** In (A) the proliferation of SR4987 in the presence of increasing concentration of PTX. The IC50 of PTX was around 34 ng/ml. In (B) is shown that viability of SR4987 is not affected by PTX even at concentration up 5000 ng/ml. In (C) is shown the growth inhibition of Molt-4 tumor cells in the presence of decreasing dilution of SR4987PTX-CM confirming that SR4987PTX release significant amount of PTX in the CM. In (D) the inhibition of proliferation of B16 cells in the presence of increasing concentration of PTX. The IC50 is 7.18 ±4.99 ng/ml. In (E) is shown the dose dependent

efficacy of SR4987PTX-CM to inhibit B16 cells proliferation with an IC50 DIL of around 1/10.

**Supplementary Figure 4**

**Co-culture of SR4987 and SR4987PTX with B16 cells using transwell insert.** B16 cells seeded on the top of membrane (0.4 um pore size) insert were co-cultured with different dose of SR4987 and SR4987PTX seeded on the bottom well and cultured for 5 days. The Figure shows that SR4987PTX at 1:5 ratio was able to strongly inhibit B16 proliferation as visualized by crystal violet staining of B16 in the transwell line (A) and on the membrane line (B). In line (C), photos of cultured B16 cells not stained upon co-culture or not with SR4987 and SR4987PTX (20x magnifications).

**Supplementary Figure 5**

**Rosette formed by B16+SR4987 and B16+SR4987PTX and their ultrastructural analysis by TEM.** In (A) and (B) rosette formed by SR4987GFP+B16 photographed under light and fluorescence microscopy respectively. (C-F) four different panels at TEM showing the interactions between untreated SR4987GFP and B16 cells. Note that B16 cells bound SR4987GFP appear healthy (C) ; the presence of area of contact between B16 and SR4987 is evident; junctional-like structure and a gap-like junction (D,E) and even nanotubule structure (F) are seen (white arrows). In (G) and (H) rosette formed by SR4987GFP-PTX+B16. Note that most of B16 cells appeared damaged and necrotic under light (G) and fluorescence (H) microscopy observation. In (I-L) four different panels at TEM showing the interaction between SR4987GFP-PTX and B16. The B16 cells bound to SR4987GFP-PTX show varying degrees of degeneration, some cells seemed even emptied of their cytoplasm (I) and, similarly to untreated SR4987, area of contact (white arrows) can be observed (J,K). In (L) a microvescicole that arise from SR4987PTX is photographed.

**Supplementary Figure 6**

**Metastasis in the lung of control mice and after treatment with PTX, SR4987 and SR4987PTX.** On day 21 after B16 cells injection, mice were euthanized, lungs removed and fixed in Bouin’s solution. In the figure photos of the lungs, taken under a dissecting stereomicroscope. Note the presence of several melanoma lung nodules (black arrows) in control and SR4987 treated mice. In contrast , at both dosage of SR4987PTX injected, the lung nodules are very few and are significantly less than those detected in PTX treated mice.

**Supplementary Figure 7**

**Chemiotaxis of SR4987 and SR4987PTX in response to B16-CM and expression of the SDF-1 receptors CXCR4 and CXCR7***.* In **(A)** migration of SR4987PTX and SR4987 placed on the top of transwell membrane insert and stimulated by different dilution of B16-CM. No significant chemotaxis activity of both SR4987 and SR4987PTX is stimulated by B16-CM at every dilutions tested. Note that the basal migration of SR4987PTX is around three fold lower than untreated SR4987 (* p<0.05). In (B) is showed FC analysis of SR4987 for the expression of the SDF-1 receptors CXCR4 and CXCR7, indicating the high positivity of the cells particularly for CXCR4 (> 90%), CXCR7 is less expressed (18%).
